# Prediction of Mechanical Properties and Fracture Behavior of TC17 Linear Friction Welded Joint Based on Finite Element Simulation

**DOI:** 10.3390/ma18010128

**Published:** 2024-12-31

**Authors:** Xuan Xiao, Yue Mao, Li Fu

**Affiliations:** 1 School of Materials Science and Engineering, Northwestern Polytechnical University, Xi’an 710072, China; featherxiao@mail.nwpu.edu.cn (X.X.); maoyue@mail.nwpu.edu.cn (Y.M.); 2 State Key Laboratory of Solidification, Northwestern Polytechnical University, Xi’an 710072, China

**Keywords:** TC17 titanium alloy, LFW, FEM, mechanical properties, fracture behavior

## Abstract

TC17 titanium alloy is widely used in the aviation industry for dual-performance blades, and linear friction welding (LFW) is a key technology for its manufacturing and repair. However, accurate evaluation of the mechanical properties of TC17−LFW joints and research on their joint fracture behavior are still not clear. Therefore, this paper used the finite element numerical simulation method (FEM) to investigate the mechanical behavior of the TC17−LFW joint with a complex micro−structure during the tensile processing, and predicted its mechanical properties and fracture behavior. The results indicate that the simulated elastic modulus of the joint is 108.5 GPa, the yield strength is 1023.2 MPa, the tensile strength is 1067.5 MPa, and the elongation is 1.98%. The deviations from measured results between simulated results are less than 2%. The stress and strain field studies during the processing show that the material located at the upper and lower edges of the joint in the WZ experiences stress and strain concentration, followed by the extending of the stress and strain concentration zone toward the center of the WZ. And finally, the strain concentration zone covered the entire WZ. The fracture behavior studies show that the material necking occurs in the TMAZ of TC17(α + β) and WZ, while cracks first appear in the WZ. Subsequently, joint cracks propagate along the TC17(α + β) side of the WZ until fracture occurs. There are obvious tearing edges formed by the partial tearing of the WZ structure in the simulated fracture surface, and there are fracture surfaces with different height differences at the center of the joint crack, indicating that the joint has mixed fracture characteristics.

## 1. Introduction

The turbines of high-performance aircraft engines mostly adopt a dual-performance integral blade structure, greatly improving their performance and safety reliability [1,2]. Linear friction welding (LFW) is a key technology for the manufacturing and repair of integral bladed disks [3,4]. Conducting basic research on LFW technology for dual-performance integral bladed disks using titanium alloys is an urgent need in the aviation industry to achieve high-performance connections of complex components, and it is also at the forefront of solid-state welding technology research [5,6].

Relevant scholars have conducted a large amount of research on micro-structure evolution, mechanical properties, fatigue properties, fracture mechanisms, and other aspects. Ma et al. [7] and Li et al. [8] studied the micro-structure and mechanical properties of the Ti17(α + β) LFW joint. They found that there were ultrafine α + β particles in WZ, and highly deformed α and β phases with specific orientations in TMAZ. The mechanical properties of the joint obtained from the test are lower than those of the base material, and the joint tends to fracture near the WZ. García et al. [9] and Ballat-Durand et al. [10] investigated the Ti17(β) LFW joint and found that there was no alpha phase in the WZ and TMAZ of the joint, thus forming a weak zone in the joint. The fatigue crack initiation and fatigue strength in this weak zone were half of those in the base metal. Ji et al. [11] found that the average yield strength and fracture strength of the dissimilar LFW joint of Ti17(α + β)-Ti17(β) were lower than those of the base materials on both sides.

Guo et al. [4,12,13] studied the micro-structure evolution, tensile properties, and impact toughness of the LFW joint of Ti17(α + β)-Ti17(β), and studied the tensile fracture mechanism of the joint through in situ tensile testing. The research results showed that due to strong thermo-mechanical coupling, complete dissolution and dynamic recrystallization of the alpha phase occurred in the WZ. This microstructural evolution led to significant work-hardening and fine-grain strengthening effects, offsetting the weakening effect caused by alpha-phase dissolution. Dyakonov et al. [14] systematically studied the micro-structure generated during the dissimilar linear friction welding process of VT8M-1 and VT25U titanium alloys. The results indicate that there is a severely deformed micro-structure and extremely strong crystallographic texture in the weld zone. And the peak welding temperature is lower than the β transus. The material flow in the α phase is mainly contributed by prismatic slip, while the material flow in the beta phase is controlled by {112} slip.

He et al. [15] discovered that the elastic modulus of TC17 alloy is positively correlated with the alpha-phase fraction, and shows a significant gradient distribution in the welding zone with a width of about 4 mm. If the gradient distribution of phase fractions is ignored, the relative error in calculating welding residual stress can reach 36.06%. The corrected residual stress in the welding area exhibits a bimodal distribution, with a peak stress of approximately 442 MPa, located at the edge of the HAZ. Jin et al. [16] studied the radial non-uniform micro-structure and properties of Ti-6Al-4V alloy rotary friction welded joint and clarified its formation mechanism through β reconstruction. Anant Sagar et al. [17] comparatively studied the strain-hardening parameters of the solid solution, aging state, and rotary friction welded joints of 1023 titanium alloy, and discussed the effect of its micro-structure on the strain-hardening behavior.

There are still certain limitations in the study of mechanical properties of the titanium alloy LFWed joint, as conventional mechanical performance tests can only obtain the average mechanical properties of the joint. However, the micro-structure of the titanium alloy LFW joint is complex, and the impact of uneven micro-mechanical properties in different areas on their macroscopic mechanical properties is not yet clear. Obtaining only average mechanical properties gradually cannot meet more refined application requirements. Meanwhile, there is no in-depth research report on the deformation law and fracture behavior of LFW joints in complex micro-structure titanium alloys.

The finite element numerical simulation method may be an effective tool for predicting the mechanical properties and fracture behavior of the LFW joint. Relevant scholars have conducted research on LFW simulation. Li et al. [18,19] established a two-dimensional numerical model and found that during the LFW cooling stage, the heat loss rate at the root of the flash is lower than the heat loss stall rate at the welding center, pointing out the importance of flash heat reflux on joint temperature. Explicit mode of the ABAQUS 6.12 software was used to conduct a two-dimensional thermo-mechanical-coupled numerical simulation of LFW of the TC4 titanium alloy, studying the influence of welding process parameters on the axial shortening and temperature evolution of the LFW joint.

Yang et al. [20] conducted a two-dimensional thermo-mechanical-coupling model on linear friction welding of TB9 titanium alloy by ABAQUS software, using the Johnson–Cook constitutive equation in the model to simulate the temperature field, von Mises stress field, and equivalent plastic strain field of the joint during LFW processing. And they simulated and experimentally verified the axial shortening amount and macroscopic morphology of the LFWed joint. McAndrew et al. [21,22,23,24] proposed a two-dimensional model of the LFW process using DEFORM 10.2 finite element software to investigate the influence of workpiece geometry on material flow, thermal field, and interface pollutant discharge during the connection process between a Ti-6Al-4V rectangular workpiece and plate.

Grujicic et al. [25] investigated the micro-structure evolution and mechanical properties of the joint. They combined the thermal–mechanical-coupling FEM simulation of the LFW process with the basic physical metallurgical laws of Ti-6Al-4V to predict the micro-structure and mechanical properties inside the LFW joint.

There have been some studies that involve the simulation of mechanical properties of the LFW joint, but most of them only focus on the temperature field changes, the micro-structure evolution, and mechanical property differences caused by micro-structure changes during the LFW process. There have been few reports on the simulation of deformation and fracture processes of the LFW joint under loading conditions. This type of research focuses on the changes in the loading process of the joint, which is of great significance for intuitively understanding the mechanical properties of LFW joints.

Therefore, this paper establishes a finite element numerical simulation model for the tensile processing of the TC17 titanium alloy LFW joint. In order to more accurately predict the mechanical properties of the joint and reproduce the tensile fracture process of the joint, the model assigns different mechanical parameters to different areas of the joint. The relevant research results can provide a new mechanical property evaluating method for a complex structured titanium alloy LFW joint, structures that cannot undergo destructive testing, and structures with high testing costs, which have significant application value.

## 2. Materials and Methods

**Figure 1:**
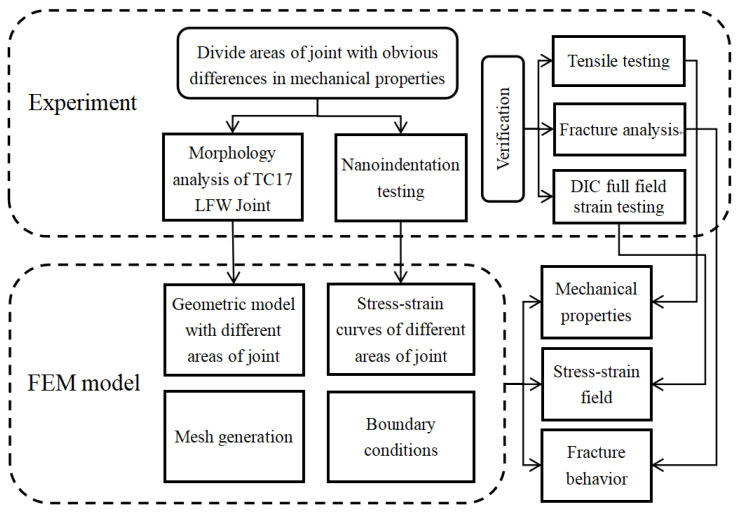
Diagram flow of the methodology and investigation steps.

The methodology and investigation steps are shown in Figure 1. Firstly, dividing the LFW joint into different areas according to the morphology analysis. Secondly, nanoindentation tests were carried out on different joint areas, and the stress–strain curves of the corresponding areas were obtained by theoretical calculation. Then, the geometric model was built according to the area division of the joint, different material parameters were set for the corresponding areas, and the boundary conditions were set according to the load in the tensile processing. Finally, the accuracy of the simulation results was verified from three aspects by tensile test, DIC full-field strain test, and fracture analysis.

### 2.1. Materials and Experimental Work

**Figure 2:**
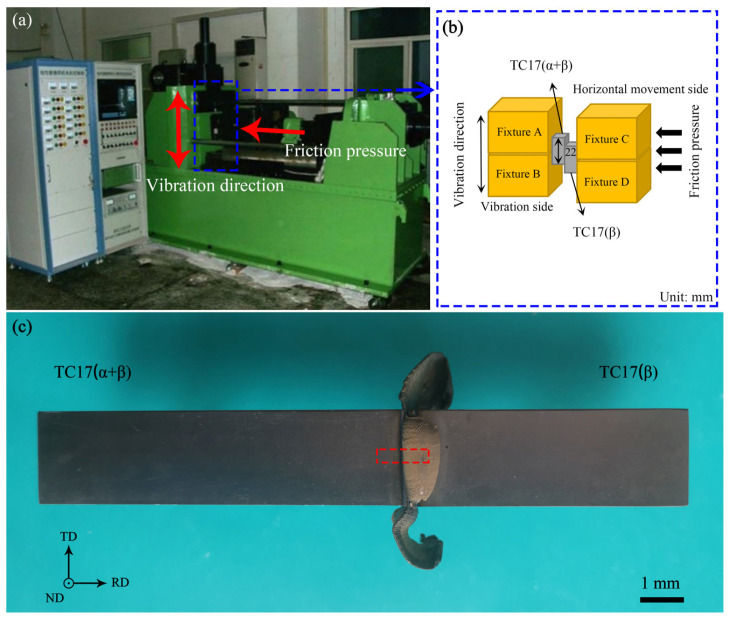
LFW machine setup and joint sample: (**a**) machine; (**b**) operation mode; (**c**) TC17−LFW joint.

The experimental materials used in this paper were two kinds of titanium alloys: TC17(α + β) and TC17(β). Table 1 shows the element composition and proportion of TC17, from the reported data [26]. The LFW process was carried out by an LFW-250 machine developed by Northwestern Polytechnic University, which can realize real-time display and control of welding process parameters (such as welding pressure, rotating speed, torque, and axial shortening). The machine setup is shown in Figure 2a,b. The welding parameters are shown in Table 2. And the linear friction welded joint of TC17 titanium alloy is shown in Figure 2c.

**Figure 3:**
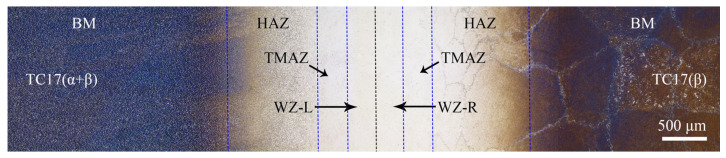
Morphology of TC17−LFW Joint.

Taking three metallographic samples with the size of 10 mm × 3 mm × 1 mm from the LFWed joint, the position is shown in the red dotted rectangular box in Figure 2c. Using Keller reagent (3 mL HCl + 5 mL HNO_3_ + 2 mL HF + 190 mL H_2_O), etch the metallographic samples after polishing. The morphology of the LFW joint was observed by an optical microscope (Olympus BX41M, Olympus Corporation, Tokyo, Japan OLYMPUS BX41M). As shown in Figure 3, the joint can be divided into the base metal zone (BM), heat-affected zone (HAZ), thermal–mechanical-affected zone (TMAZ), and welding zone (WZ) based on the differences in micro-structure. While the WZ can be divided into WZ-L and WZ-R based on their different micro-mechanical properties [26], which represent the TC17(α + β) and TC17(β) sides of the WZ, respectively.

Nanoindentation testing was conducted on the TC17−LFW joint using an indentation loading force of 1.96 N. Briefly, 5 points in each of the 7 different areas of the joint were tested, and the average of the testing results was taken. Subsequently, based on the principle of inverse material parameters using load–displacement curves [27,28,29,30,31,32,33], the corresponding material constitutive relationships in different areas of the joint were calculated and obtained.

**Figure 4:**
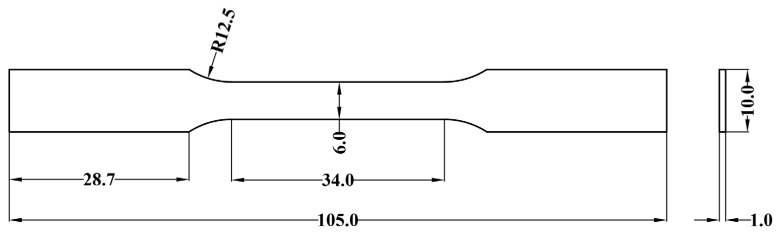
Schematic diagram of tensile specimen.

The three tensile samples were prepared according to standard GBT-228.1-2021 [34], whose dimension data are shown in Figure 4. The gauge area of the specimen was polished to 800 # along the loading direction with SiC sandpaper to obtain a smooth surface. The tensile testing was carried out with a universal testing machine (GNT100, Steel Research Nak Testing Technology Co., Ltd., Beijing, China GNT100), with a cross-head speed of 1 mm/min. An electronic extensometer was used, which has a gauge length of 25 mm. The average of the testing results was taken from the three samples.

A non-contact digital image correlation (DIC) full-field strain test system (VIC-3D) was used during the standard tensile testing. The strain distribution of the sample in the tensile processing was obtained through this system, which provides an experimental basis for studying the evolution law of the strain field.

### 2.2. Finite Element Model of Tensile Processing

In this paper, a 3D mathematical model of tensile processing of the TC17−LFW joint was developed using commercial FEM software ABAQUS to investigate the joint’s mechanical properties, stress and strain field, deform and fracture process. Before the simulation, some assumptions and simplifications were put forward to the model.

The model was constructed based on standard tensile specimens, the clamping section can be simplified by shorting its length to reduce the computational cost of the model. Explicit mode was adopted to accurately simulate the crack propagation during the fracture process. Then, the software parameters were set as follows: the calculation mode is standard & explicit, the initial step size is 0.1, and the mass scaling is 10^−6^, others are default values.

#### 2.2.1. Geometric Model and Mesh Generation

**Figure 5:**
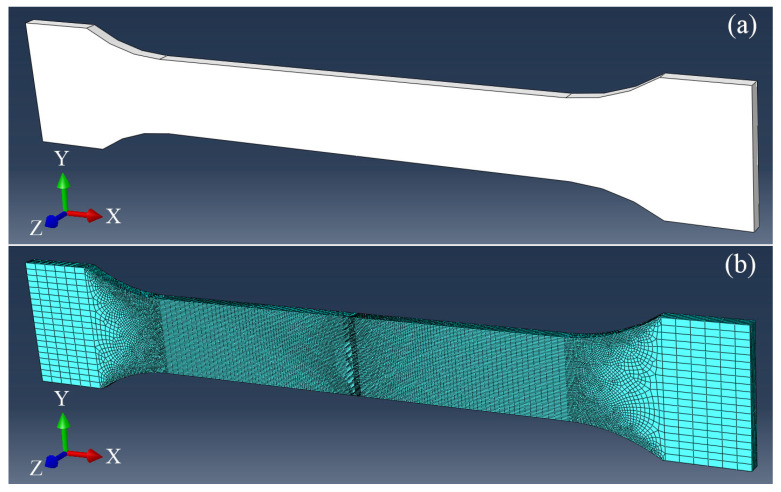
Geometric model and mesh of tensile processing in TC17−LFW joint: (**a**) geometric model; (**b**) mesh.

The geometric model constructed based on the standard tensile specimen of the LFWed joint is shown in Figure 5a, while the structural dimensions are shown in Figure 4. The length of the parallel section is 34 mm, the length of the clamping section is set to 10 mm, and the weld is located in the center of the parallel section. Figure 5b shows the schematic diagram of the model mesh division. The model adopts a hexahedral C3D8R mesh, where the sizes of the global mesh element, weld zone mesh element, and the clamping section mesh element are set to 0.1, 0.05, and 1, respectively. The total number of meshes is 44,982.

#### 2.2.2. Material Mechanical Properties

**Figure 6:**
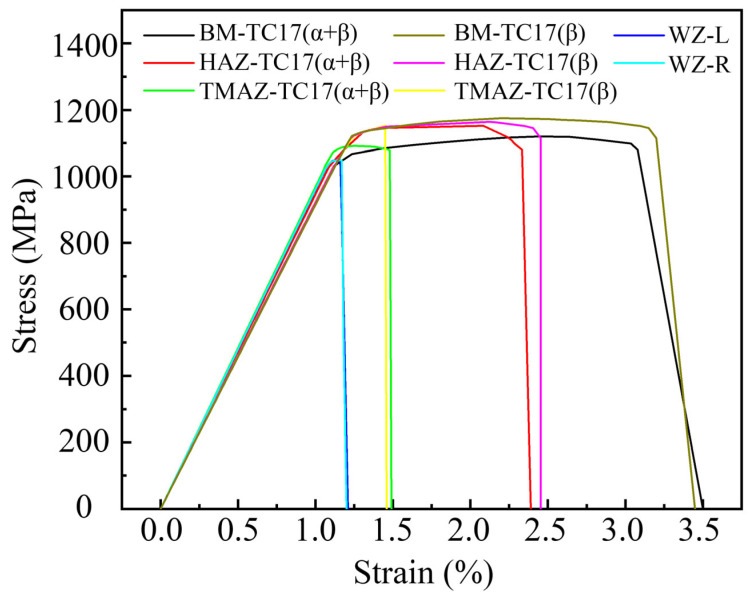
Stress–strain curves of different areas of TC17−LFW joint obtained by inverse material parameters using nanoindentation load displacement curves.

Figure 6 shows stress–strain curves of different areas of the TC17−LFW joint obtained by inverse material parameters using nanoindentation load displacement curves [24]. The elastic modulus, yield strength, tensile strength, and elongation of BM of TC17(α + β) are 92.3 GPa, 1030.0 MPa, 1119.7 MPa, and 3.50%, respectively. The elastic modulus, yield strength, tensile strength, and elongation of HAZ of TC17(α + β) are 94.6 GPa, 1025.7 MPa, 1152.0 MPa, and 2.49%, respectively. The elastic modulus, yield strength, tensile strength, and elongation of TMAZ of TC17(α + β) are 96.9 GPa, 1021.4 MPa, 1092.0 MPa, and 1.49%, respectively. The elastic modulus, yield strength, tensile strength, and elongation of WZ-L are 96.0 GPa, 1015.6 MPa, 1049.4 MPa, and 1.21%, respectively. The elastic modulus, yield strength, tensile strength, and elongation of WZ-R are 96.0 GPa, 1016.8 MPa, 1049.0 MPa, and 1.20%, respectively. The elastic modulus, yield strength, tensile strength, and elongation of TMAZ of TC17(β) are 92.31 GPa, 1027.3 MPa, 1153.0 MPa, and 1.46%, respectively. The elastic modulus, yield strength, tensile strength, and elongation of HAZ of TC17(β) are 92.0 GPa, 1029.5 MPa, 1164.3 MPa, and 2.45%, respectively. The elastic modulus, yield strength, tensile strength, and elongation of BM of TC17(β) are 91.6 GPa, 1031.8 MPa, 1175.5 MPa, and 3.45%, respectively.

**Figure 7:**
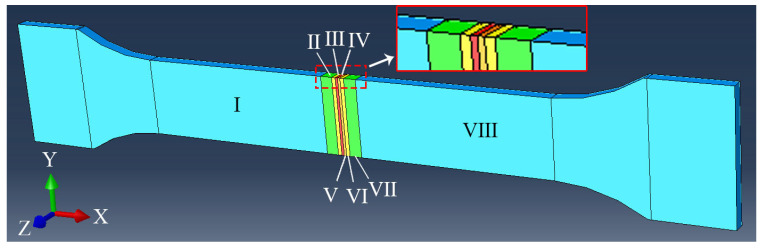
Material parameter settings of tensile processing model in TC17−LFW joint.

Figure 7 shows a schematic diagram of the material parameter settings of the tensile processing model in the TC17−LFW joint. Among them, I is the BM of TC17(α + β), II is the HAZ of TC17(α + β), III is the TMAZ of TC17(α + β), IV is the TC17(α + β) side of the WZ, V is the TC17(β) side of the WZ, VI is the TMAZ of TC17(β), VII is the HAZ of TC17(β), and VIII is the BM of TC17(β). The material parameters in different areas are the stress–strain curves in Figure 5.

#### 2.2.3. Boundary Conditions

**Figure 8:**
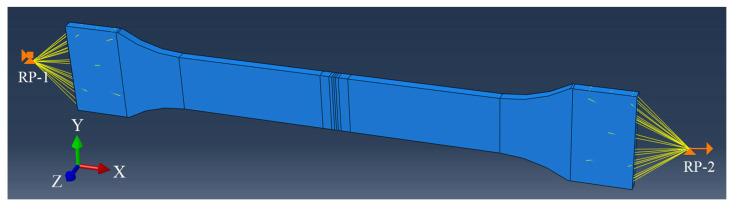
Boundary conditions of tensile processing model in TC17−LFW joint.

As shown in Figure 8, there are 2 reference points set on both sides of the model. The RP-1 point is coupled with the surfaces of the clamping section. The RP-2 point is coupled with the surfaces of the mobile section. Then, the RP-1 point is set as a fully fixed constraint. The RP-2 point is set to constraints in the Y and Z directions, as well as a displacement of 10 mm in the X positive direction. These boundary condition settings can accurately simulate the loading state of the specimen during the tensile processing.

## 3. Results and Discussion

### 3.1. Mechanical Properties

**Figure 9:**
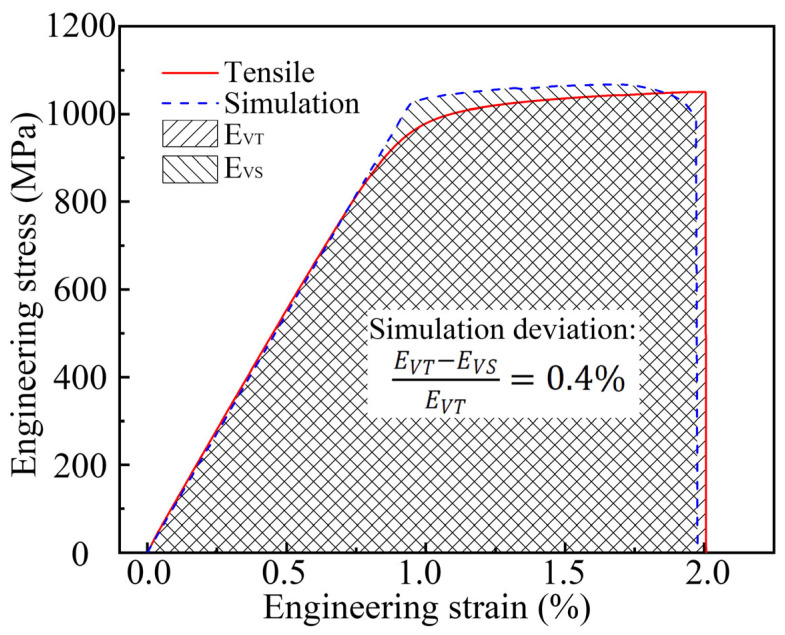
Boundary conditions of tensile processing model in TC17−LFW joint.

The engineering stress–strain curve of the tensile processing of the TC17−LFW joint is shown in Figure 9. The red solid line represents the results obtained from conventional tensile testing, while the blue dashed line represents the simulation calculation results obtained through the homogenization method. The mechanical properties of the joint obtained by taking the average of the conventional tensile test results are as follows: the elastic modulus is 109.9 GPa, the yield strength is ~1003.5 MPa, the tensile strength is 1050.3 MPa, and the elongation is ~2.01%. The simulated elastic modulus, yield strength, tensile strength, and elongation of the joint are 108.5 GPa, 1023.2 MPa, 1067.5 MPa, and 1.98%, respectively. The deviation rates of the above mechanical properties indicators are elastic modulus 1.3%, yield strength 1.9%, tensile strength 1.6%, and elongation 1.5%.

The area enclosed under the stress–strain curve can represent the energy absorbed per unit volume of material. The value of the area under the experimental test curve E_VT_ is 1574.9, while the value of the area under the simulated curve E_VS_ is 1567.9, with a deviation rate of 0.4%, indicating that the numerical simulation results are relatively accurate. This model can be used to conduct detailed research on the stress–strain distribution, deformation, and fracture behavior during tensile processing.

### 3.2. Stress–Strain Field

**Figure 10:**
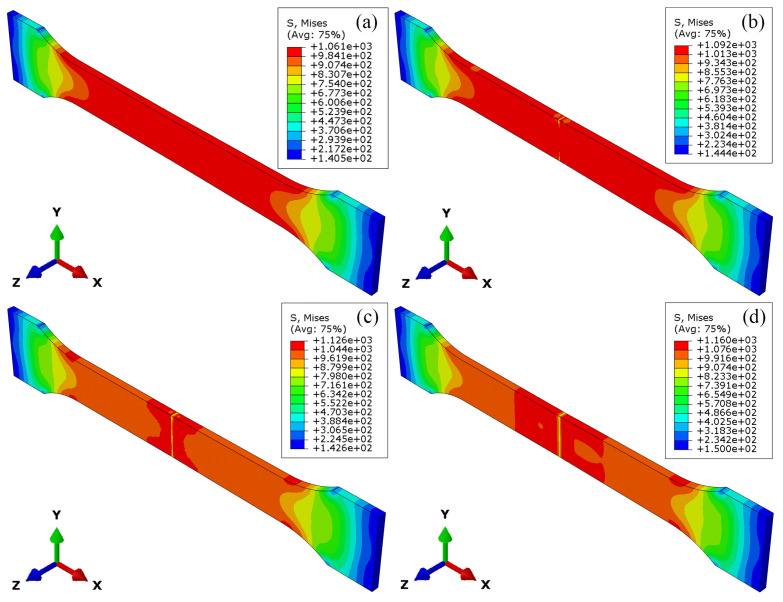
Stress field during tensile processing of TC17−LFW joint: (**a**) after yielding; (**b**) deformed 45%; (**c**) deformed 60%; (**d**) Deformed 90%.

**Figure 11:**
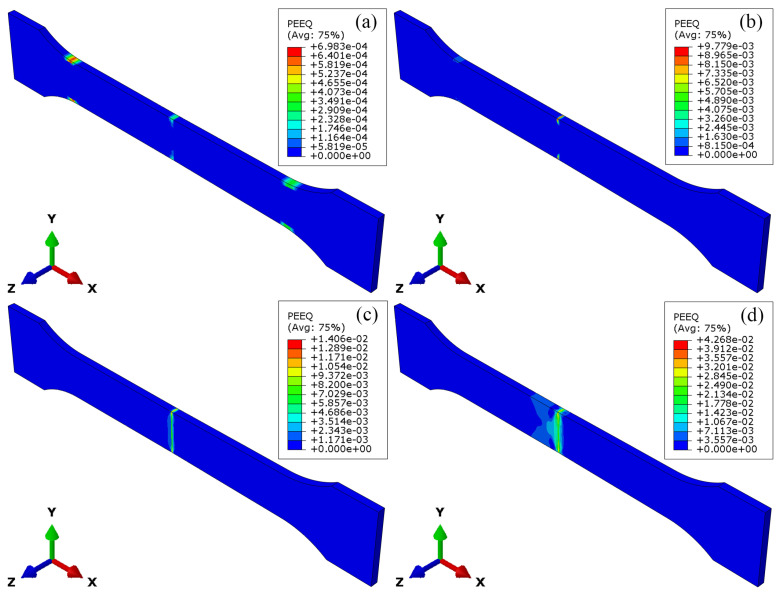
Strain field during tensile processing of TC17−LFW joint: (**a**) after yielding; (**b**) deformed 45%; (**c**) deformed 60%; (**d**) deformed 90%.

The stress field and strain field during the tensile processing of the LFW joint are shown in Figure 10 and Figure 11, respectively. According to Figure 10a, when the joint yields, the peak stress is 1061 MPa. At this point, plastic strain begins to appear in the parallel section and the end of the arc-shaped transition section of the specimen, as shown in Figure 11a. When the joint deforms by 45%, the peak stress of the joint is 1092 MPa. The material stress at the upper and lower edges of the WZ of the joint is lower than that in other areas of the joint, and the strain at this position is significantly higher than that in other areas of the joint, as shown in Figure 10b and Figure 11b.

When the joint deforms by 60%, the peak stress of the joint is 1126 MPa. The low-stress area in the WZ extends toward the center of the joint, and strain concentration begins to appear in the WZ, as shown in Figure 10c and Figure 11c. When the joint deforms by 90%, the peak stress of the joint is 1160 MPa, and the range of low-stress area expands to include the entire WZ and TMAZ of TC17(α + β). The strain concentration range of the WZ extends to TMAZ of TC17(α + β) and HAZ of TC17(α + β), as shown in Figure 10d and Figure 11d.

**Figure 12:**
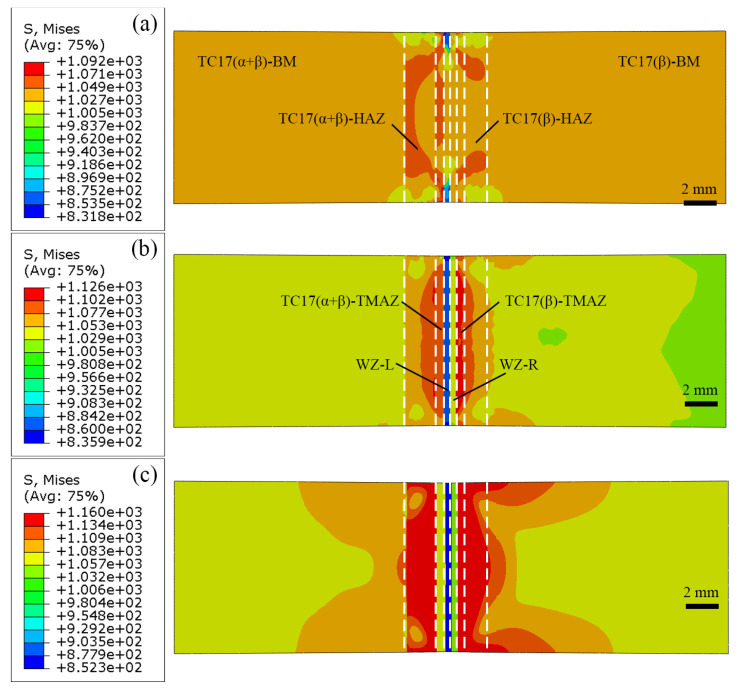
Stress field during tensile processing of surface XOY of TC17−LFW joint: (**a**) deformed 45%; (**b**) deformed 60%; (**c**) deformed 90%.

**Figure 13:**
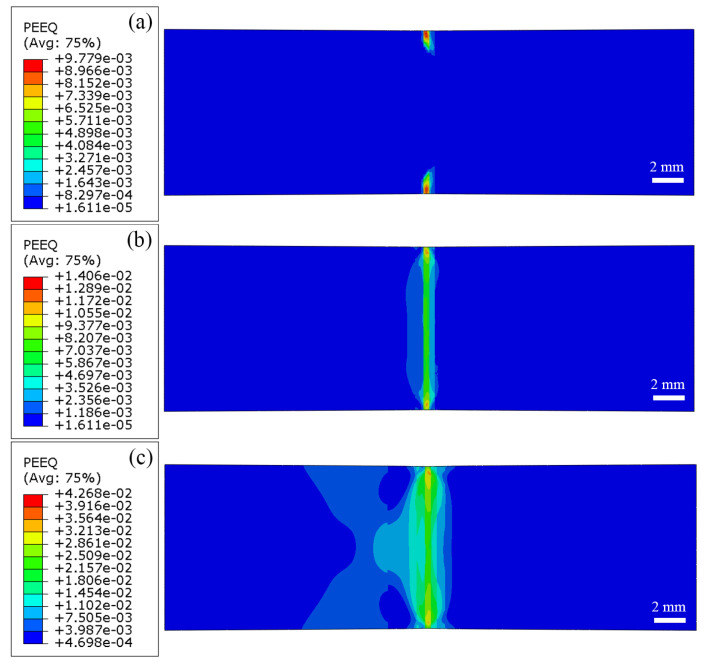
Strain field during tensile processing of surface XOY of TC17−LFW joint: (**a**) deformed 45%; (**b**) deformed 60%; (**c**) deformed 90%.

The stress field and strain field of the XOY surface during the tensile processing of the LFW joint are shown in Figure 12 and Figure 13, respectively, the parallel section of the joint is selected for research and analysis. From Figure 12a and Figure 13a, when the joint deforms by 45%, the stress in the parallel section of the joint is between 832 MPa and 1092 MPa, and the strain is between 1.61 × 10^−5^ and 9.78 × 10^−3^. At this time, the low-stress zone (i.e., the strain concentration zone) is located on the upper and lower edges of the TC17(α + β) side of the WZ (WZ-L), indicating that the material on the TC17(α + β) side of the WZ has begun to show damage.

When the joint deforms by 60%, the stress in the parallel section of the joint is between 836 MPa and 1126 MPa, and the strain is between 1.61 × 10^−5^ and 1.41 × 10^−2^. At this time, the low-stress zone is located on the TC17(α + β) side of the entire WZ (the entire WZ-L) of the joint, and the strain concentration zone is the entire WZ, as shown in Figure 12b and Figure 13b. When the joint deforms by 90%, the stress in the parallel section of the joint is between 852 MPa and 1160 MPa, and the strain is between 4.70 × 10^−4^ and 4.27 × 10^−2^. The low-stress area extends to cover the entire WZ and TC17 (α + β)-TMAZ. The stress concentration range on both sides of the WZ increases, and the strain concentration zone extends to the entire WZ, as shown in Figure 12c and Figure 13c.

**Figure 14:**
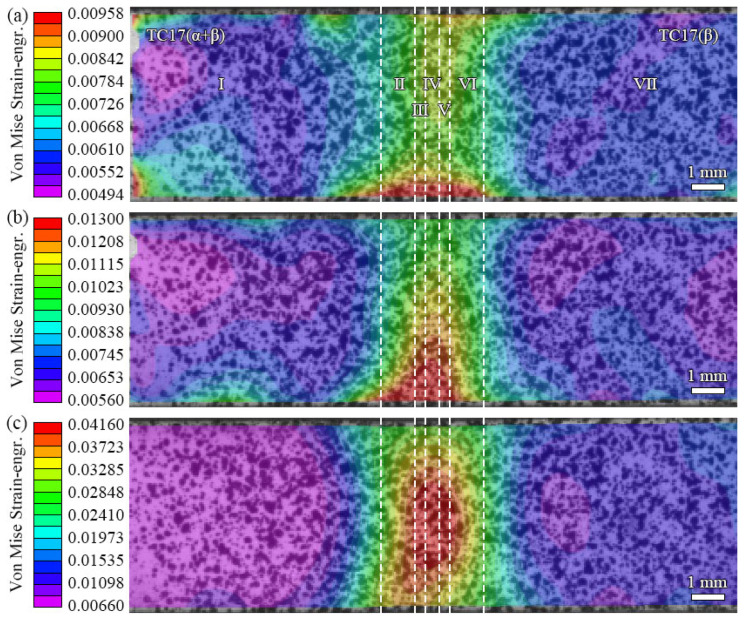
DIC full-field strain during tensile processing of TC17−LFW joint: (**a**) deformed 45%; (**b**) deformed 60%; (**c**) deformed 90%.

Figure 14 shows the DIC full-field strain during the tensile processing of the TC17−LFW joint. According to Figure 14a, when the joint deforms by 45%, the strain of the parallel section of the joint is between 4.94 × 10^−3^ and 9.58 × 10^−3^, and its upper limit is close to the simulated 9.78 × 10^−3^. At the same time, it can be seen that the strain concentration zone is located at the lower edge of the WZ of the joint. According to Figure 14b, when the joint deforms by 60%, the strain of the parallel section of the joint is between 5.60 × 10^−3^ and 1.30 × 10^−2^, and its upper limit is close to the simulated value of 1.41 × 10^−2^. The strain concentration zone extends from the lower edge of the WZ toward the center of the joint. According to Figure 14c, when the joint deforms by 90%, the strain of the parallel section of the joint is between 6.60 × 10^−3^ and 4.16 × 10^−2^, and its upper limit is close to the simulated 4.27 × 10^−2^; at this point, the strain concentration zone extends to the entire WZ, and the peak is located at the centerline of the joint.

The above phenomenon indicates that the strain field evolution during the simulated LFW joint tensile processing is basically consistent with the experimental strain field evolution process. The strain values of the two are close under different deformation amounts, and the distribution and variation law of the strain concentration zone are similar. The only difference is that the simulated strain concentration zone changes from both sides of the upper and lower edges of the joint WZ toward the center of the joint, while the strain concentration zone of the experimental specimen changes from one side of the lower edge of the joint WZ toward the center of the joint. This is because the model is an ideal simplified symmetrical model, while the specimen may not be completely consistent in the state of the upper and lower edges of the WZ due to the influence of its preparation process, causing the phenomenon of only one-sided strain concentration in the specimen mentioned above.

### 3.3. Fracture Behavior

**Figure 15:**
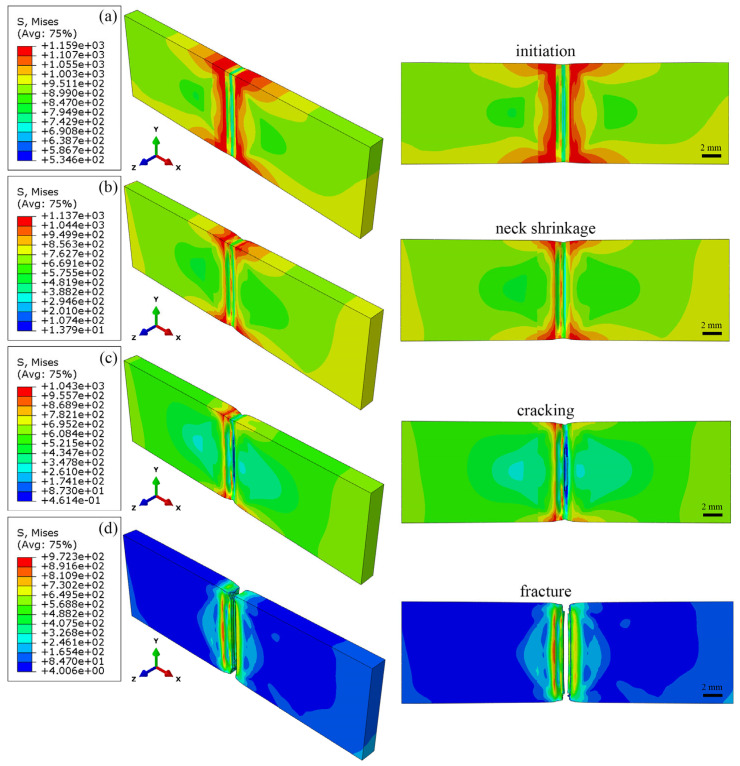
Neck shrinkage deformation and fracture behavior during tensile processing of TC17−LFW joint: (**a**) initiation; (**b**) neck shrinkage; (**c**) cracking; (**d**) fracture.

Figure 15 shows the necking deformation and fracture behavior of the TC17−LFW joint during the tensile processing. As shown in Figure 15a, stress concentration occurs on both sides of the WZ at the beginning of necking, and the material in the WZ begins to be damaged, resulting in a decrease in stress. According to Figure 15b, as the stress in the WZ further decreases, significant necking occurs both in the TMAZ of TC17(α + β) and WZ, and the material in the WZ deforms along the Y and Z directions at the center of the joint. As shown in Figure 15c, cracks first appear in the WZ, while the stress concentration area shrinks, only partially existing on the TC17(α + β) side of the WZ. Subsequently, the joint crack propagated along the TC17(α + β) side of the WZ until fracture occurred, as shown in Figure 15d.

**Figure 16:**
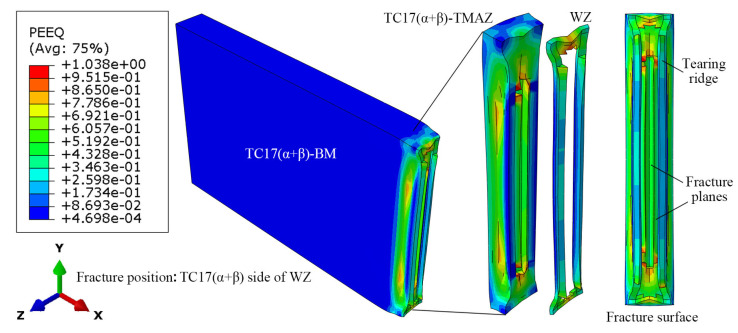
Simulation fracture morphology of TC17−LFW joint.

The fracture morphology of the TC17−LFW joint after simulated tensile fracture is shown in Figure 16. There is a significant necking phenomenon in the TMAZ of TC17(α + β) and WZ, with the fracture located on the TC17(α + β) side of the WZ. During fracture, some WZ structures were torn to form obvious tearing edges. And there were fracture surfaces with different height differences at the center of the joint crack. Some sections were even located in the TMAZ of TC17(α + β). Due to significant differences in structures of different areas, the fracture mode presented by the joint section is a mixed fracture.

**Figure 17:**
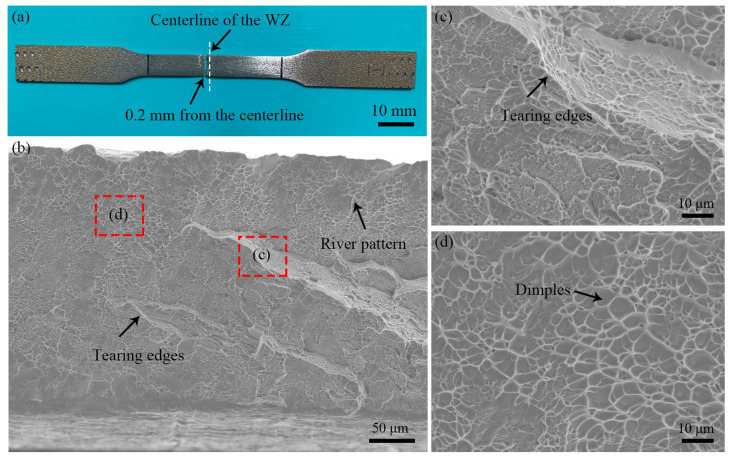
Fracture morphology of TC17−LFW joint: (**a**) overall morphology; (**b**–**d**) local fracture morphology.

As shown in Figure 17a, the joint fracture occurred on the TC17(α + β) side of the WZ zone (at a distance of 0.2 mm from the centerline of the welding zone), indicating that the simulated predicted fracture location is consistent with the actual fracture location. Meanwhile, as shown in Figure 17b–d, the fracture surface of the TC17−LFW joint exhibits characteristics of ductile dimples, large and continuous tearing edges, river pattern, and fracture surfaces with varying heights, collectively indicating that the tensile fracture mode of the TC17−LFW joint is a mixed fracture mode.

## 4. Conclusions

In this paper, the stress–strain evolution, deformation law, and fracture behavior of TC17−LFW joint in the tensile processing are clarified by combining experimental and simulated research and analysis. The main conclusions are as follows:

(1) The simulated mechanical properties of TC17−LFW joint are as follows: elastic modulus of 108.5 GPa, yield strength of 1023.2 MPa, tensile strength of 1067.5 MPa, and elongation of 1.98%, with deviations from measured results of less than 2%.
(2) The material located at the upper and lower edges of the joint in the WZ first experiences stress and strain concentration, followed by the extending of the stress and strain concentration zone toward the center of the WZ. And finally, the strain concentration zone covered the entire WZ.
(3) Material necking occurs in the TMAZ of TC17(α + β) and WZ, while cracks first appear in the WZ. Subsequently, joint cracks propagate along the TC17(α + β) side of the WZ until fracture occurs.
(4) There are obvious tearing edges formed by the partial tearing of the WZ structure in the simulated fracture surface, and there are fracture surfaces with different height differences at the center of the joint crack, indicating that the joint has mixed fracture characteristics.

The relevant research methods and results have important application value in the evaluation of mechanical properties of structures with a complex micro-structure, but the value in the mechanical properties of homogeneous materials or structures is limited. The potential areas of future research are mainly in investigating mechanical properties of the structures that cannot be subjected to destructive tests or have high test costs, or in extending the scope of application to a simulation evaluation of other performance.

## Figures and Tables

**Table 1:** Element composition and proportion of TC17 titanium alloy (wt,%).

Base Metal	Al	Sn	Zr	Mo	Cr	Fe	Ti
TC17(α + β)	5.07	2.06	2.05	3.80	3.99	0.15	Bal
TC17(β)	5.1	2.0	1.9	4.1	3.9	0.04	Bal

**Table 2:** The welding parameters of TC17−LFW processing.

Vibration Frequency	Vibration Amplitude	Friction Pressure	Upset Pressure
50 Hz	2 mm	55 MPa	110 MPa

## Data Availability

The original contributions presented in this study are included in the article. Further inquiries can be directed to the corresponding author.
